# A selective sweep of >8 Mb on chromosome 26 in the Boxer genome

**DOI:** 10.1186/1471-2164-12-339

**Published:** 2011-07-01

**Authors:** Javier Quilez, Andrea D Short, Verónica Martínez, Lorna J Kennedy, William Ollier, Armand Sanchez, Laura Altet, Olga Francino

**Affiliations:** 1 Molecular Genetics Veterinary Service (SVGM), Department of Animal and Food Science, Veterinary School, Universitat Autònoma de Barcelona (UAB), 08193 Bellaterra, Barcelona, Spain; 2 Centre for Integrated Genomic Medical Research (CIGMR), Stopford Building, Oxford Road, Manchester, M13 9PT, UK

## Abstract

**Background:**

Modern dog breeds display traits that are either breed-specific or shared by a few breeds as a result of genetic bottlenecks during the breed creation process and artificial selection for breed standards. Selective sweeps in the genome result from strong selection and can be detected as a reduction or elimination of polymorphism in a given region of the genome.

**Results:**

Extended regions of homozygosity, indicative of selective sweeps, were identified in a genome-wide scan dataset of 25 Boxers from the United Kingdom genotyped at ~20,000 single-nucleotide polymorphisms (SNPs). These regions were further examined in a second dataset of Boxers collected from a different geographical location and genotyped using higher density SNP arrays (~170,000 SNPs). A selective sweep previously associated with canine brachycephaly was detected on chromosome 1. A novel selective sweep of over 8 Mb was observed on chromosome 26 in Boxer and for a shorter region in English and French bulldogs. It was absent in 171 samples from eight other dog breeds and 7 Iberian wolf samples. A region of extended increased heterozygosity on chromosome 9 overlapped with a previously reported copy number variant (CNV) which was polymorphic in multiple dog breeds.

**Conclusion:**

A selective sweep of more than 8 Mb on chromosome 26 was identified in the Boxer genome. This sweep is likely caused by strong artificial selection for a trait of interest and could have inadvertently led to undesired health implications for this breed. Furthermore, we provide supporting evidence for two previously described regions: a selective sweep on chromosome 1 associated with canine brachycephaly and a CNV on chromosome 9 polymorphic in multiple dog breeds.

## Background

It has been proposed that the majority of modern dog breeds recognised today have resulted from two population bottlenecks in dog evolution [1,2]. During the first genetic bottleneck, pre-domestic breeds diverged from wolves some 15,000 years ago, probably through multiple domestication events. The second bottleneck for most breeds occurred within the last few hundred years, when the breed creation process resulted in the loss of genetic variation due to strong bottleneck events which occurred in parallel with strong artificial selection for behavioural and physical characteristics favoured by humans.

The same bottlenecks and artificial selection forces that generated these breed-specific features have, in some instances, provoked undesired health effects. Random fixation of detrimental variants can occur during bottlenecks. Similarly, risk alleles may be in linkage disequilibrium with selected phenotypic variants or these may have pleiotropic effects [3,4].

Several studies have previously aimed to identify genomic regions involved in defined traits and their relationship with disease using association mapping (reviewed in Karlsson and Linblad-Toh [2]). However, phenotypic traits that have been driven to fixation by genetic drift or artificial selection within a dog breed cannot be mapped within that breed with this approach. An alternative in these cases is selection mapping, in which selective sweeps (a reduction or elimination of genetic polymorphism in a region owing to strong selection) are searched [2,5-8]. The aim of this work was to identify selective sweeps in the Boxer genome resulting from the breed creation process using high density genome-wide SNP data. These regions are likely to govern phenotypic traits of interest and may be linked to overrepresentation of certain genetic disorders in this breed.

## Results

### Detection and replication in Boxer

**Figure 1:**
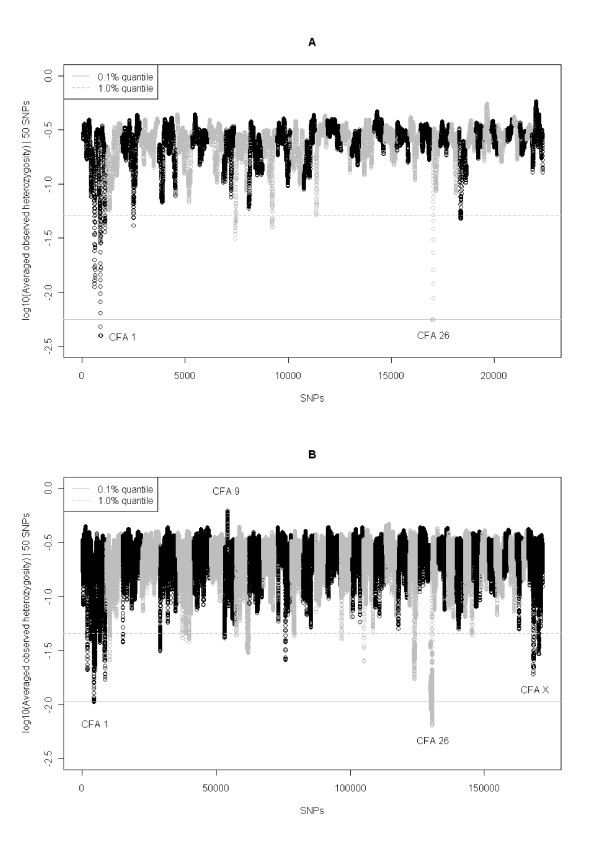
**Genome-wide plot of the averaged observed heterozygosity with the 50-SNP sliding window for set A (a) and set B (b)**. The x-axis corresponds to SNPs sort by chromosomes (differently coloured) and position and the y-axis represents the 10-logarithm of the averaged observed heterozygosity calculated with sliding windows of 50 SNPs, for which 0.1 and 1.0% quantiles of the empirical distribution are displayed.

**Figure 2:**
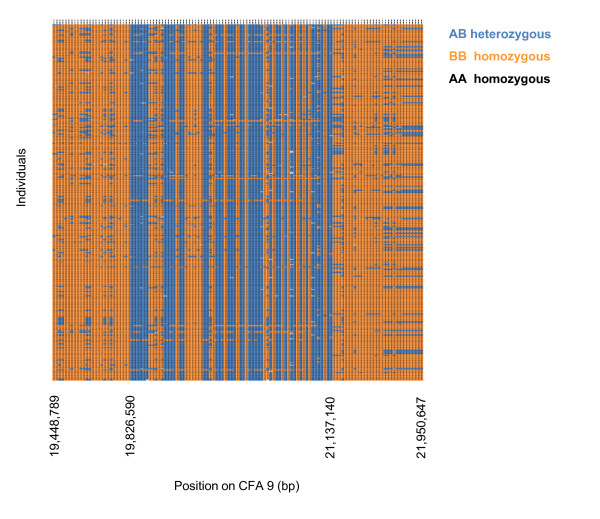
**Pattern of alternate heterozygous/homozygous genotypes in a 1.5-Mb region corresponding to a described CNV on CFA 9**. In the x-axis, the start and end positions of both the chromosome region shown and the CNV are indicated.

Regions of homozygosity (ROHs) in the *Canis familiaris *chromosomes (CFA) were identified in 25 Boxers from the United Kingdom (UK) which had been genotyped on microarrays for ~20,000 SNPs (set A, Table 1). Eight ROHs meeting the criteria detailed in Methods were detected (Table 2), representing 22 Mb (~0.9%) of the dog genome. Three of these ROHs, two on CFA 1 and one on CFA 26, showed a remarkable extended low heterozygosity (Figure 1a, Table 2). To confirm these ROHs, a second dataset (set B, Table 1) was generated using a higher density SNP array (~170,000 SNPs). This related to Boxers collected from different geographical locations to set A. In set B, 27 ROHs were found, which spanned 40.8 Mb (~1.7%) of the dog genome. Three regions on CFA X (Figure 1b) were discarded as these were not present when only female samples were analyzed (data not shown). Five ROHs were shared in both sets (Table 2). In general, these were notably shorter and/or split into two separated shorter regions when a higher number of samples and SNPs were genotyped (Additional file 1: Figure S1a-d, f-i). Conversely, the first 8 Mb of CFA 26 showed almost total loss of heterozygosity (average observed marker heterozygosity < 0.02) in both sets (Additional file 1: Figure S1e, j). There was a single SNP (BICF2G630807104, CFA 26:4,222,068 bp) with MAF = 0.5 within the region of extended homozygosity on CFA 26 (Additional file 1: Figure S1j), closer examination of which showed that heterozygous genotypes had been called for all Boxer samples. Possible explanations might be wrong genotype call from intensity data or a structural variation affecting that single SNP. To avoid the concern about SNPs significantly deviating from Hardy-Weinberg Equilibrium (HWE) affecting the identification of ROHs the analysis was repeated in set B after the removal of SNPs with HWE test p-value < 0.005, which resulted in similar results (Additional file 2 and Additional file 3). For the subsequent analyses we focused on the ROHs on CFA 1:58,710,420-61,801,815 bp and CFA 26:3,008,718-11,914,284 bp because these had markedly larger size and lower levels of variation than other regions common in both sets (Figure 1, Table 2). Finally, we found a region of increased heterozygosity on CFA 9:19,826,590-21,137,140 bp (Figure 1b), closer examination of which revealed a region of approximately 1.5 Mb showing a pattern of alternate heterozygous and homozygous genotypes indicating a CNV (Figure 2).

**Table 1:** Samples genotyped with call rate > 90%.

Group	# Samples	BeadChip
Boxer (denoted as set A)	25	Illumina's CanineSNP20 (~20,000 SNPs)
Iberian wolf	7	
Shar pei	37	
Cirneco dell'Etna	12	
Canarian warren hound	13	
Ibizan hound	39	
Pharaoh hound	5	

Labrador retriever^1^	12	Illumina's CanineHD (~170,000 SNPs)
Boxer (denoted as set B)	273	
German shepherd dog (GSD)	43	
English bulldog	4	
French bulldog	6	
Pug	10	

^1^a subset of ~17,000 SNPs shared with the Illumina's CanineSNP20 was used for this breed.

**Table 2:** Regions of homozygosity (ROHs) detected in sets A and B.

*Set A*								
**Region ID**	**CFA**	**BP1**	**BP2**	**KB**	**NSNP**	**KB/SNP**	**Avg. Het**	**Group**

SetA_01	1	57,121,838	67,829,167	10,707	84	127.5	0.02	*1
SetA_02	1	86,903,752	95,179,544	8,276	90	92.0	0.03	*2
SetA_03	1	112,558,604	120,632,074	8,073	71	113.7	0.05	*3
SetA_04	3	62,679,327	67,735,724	5,056	53	95.4	0.05	
SetA_05	10	3,081,933	12,082,601	9,001	68	132.4	0.04	*4
SetA_06	12	48,950,221	55,687,381	6,737	63	106.9	0.04	
SetA_07	26	3,116,745	12,410,004	9,293	107	86.9	0.01	*5
SetA_08	29	11,263,518	18,670,911	7,407	69	107.4	0.05	

*Set B*								

**Region ID**	**CFA**	**BP1**	**BP2**	**KB**	**NSNP**	**KB/SNP**	**Avg. Het**	**Group**

SetB_01	1	26,672,978	27,730,188	1,057	78	13.6	0.03	
SetB_02	1	45,218,029	46,286,798	1,069	66	16.2	0.04	
SetB_03	1	58,732,954	61,801,815	3,069	221	13.9	0.03	*1
SetB_04	1	62,722,220	65,190,321	2,468	129	19.1	0.03	*1
SetB_05	1	89,187,131	90,230,941	1,044	86	12.1	0.04	*2
SetB_06	1	102,454,189	103,320,473	866	51	17.0	0.04	
SetB_07	1	116,688,554	118,107,497	1,419	97	14.6	0.03	*3
SetB_08	1	117,979,514	118,963,939	984	51	19.3	0.04	*3
SetB_09	2	22,715,411	24,024,566	1,309	78	16.8	0.04	
SetB_10	3	3,030,299	3,903,071	873	59	14.8	0.04	
SetB_11	5	4,697,408	6,247,457	1,550	105	14.8	0.04	
SetB_12	6	25,815,666	26,601,998	786	52	15.1	0.04	
SetB_13	6	42,437,711	43,955,158	1,517	60	25.3	0.04	
SetB_14	6	58,580,737	59,356,441	776	62	12.5	0.04	
SetB_15	9	3,529,583	4,304,182	775	58	13.4	0.04	
SetB_16	10	5,626,769	6,702,961	1,076	51	21.1	0.04	*4
SetB_17	10	59,180,359	60,456,532	1,276	90	14.2	0.04	
SetB_18	10	65,209,901	66,606,742	1,397	86	16.2	0.04	
SetB_19	10	68,323,860	69,026,432	703	61	11.5	0.04	
SetB_20	13	39,705,171	40,797,838	1,093	71	15.4	0.04	
SetB_21	14	19,806,989	20,470,904	664	52	12.8	0.04	
SetB_22	18	6,868,787	7,908,159	1,039	54	19.2	0.04	
SetB_23	20	7,816,139	8,635,017	819	58	14.1	0.03	
SetB_24	24	24,444,170	27,387,620	2,943	217	13.6	0.03	
SetB_25	24	28,845,341	29,848,829	1,003	93	10.8	0.03	
SetB_26	26	3,008,718	11,914,284	8,906	707	12.6	0.01	*5
SetB_27	30	38,126,268	38,689,821	564	52	10.8	0.04	

CFA, *Canis familiaris *chromosome; BP1 and BP2, base pair positions of the first and last SNP involved in the ROH, respectively; NSNP, number of SNPs involved in the ROH; KB/SNP, SNP density in the ROH; Avg. Het, average observed heterozygosity. ROHs present in both sets are grouped numerically. Note that the end of SetB_07 overlaps with start of SetB_08.

### Presence in other breeds

**Figure 3:**
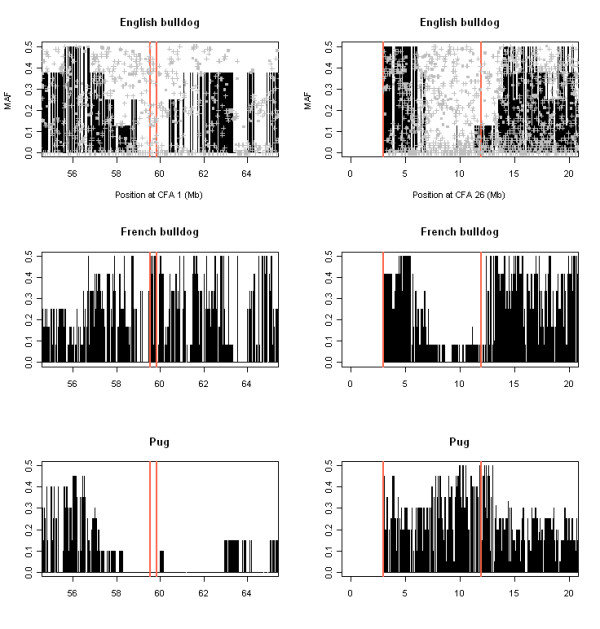
**ROHs on CFA 1 and CFA 26 in non-brachycephalic and brachycephalic breeds other than Boxer**. In the top panels, MAF values for the pool of non-brachycephalic breeds (grey squares) and German shepherd dog (grey crosses) are shown. For CFA 1, the region associated with brachycephaly by Bannasch *et al. *(CFA 1:59,536,208-59,832,965 bp) [7] is indicated (red lines). For CFA 26, the red lines show the extent of the ROH in Boxer for comparison with the other breeds.

Since reduction of genetic polymorphism in a region can result from strong selection and brachycephaly is a breed-defining trait in the Boxer, we evaluated the presence of the ROHs on CFA 1 and 26 in non-brachycephalic and brachycephalic breeds. Brachycephaly is characterized by severe shortening of the muzzle, and therefore the underlying bones, and a more modest shortening and widening of the skull [9]. For both selective sweeps on CFAs 1 and 26, normal levels of heterozygosity were observed in non-brachycephalic dog breeds and the Iberian wolf (Figure 3), based on a first dataset containing 118 samples from 6 different dog breeds and 7 Iberian wolf samples genotyped using the same panel of SNPs as in set A and on a second dataset containing 43 samples from German shepherd dog genotyped using the same panel of SNPs as in set B (Table 1).

The selective sweep on CFA 1 was present and showed allelic match with the Boxer (data not shown) in other brachycephalic breeds such as English bulldog, Pug and French bulldog although in the latter the reduction in heterozygosity was not as extended as in the other two breeds and seemed to be located slightly upstream in the chromosome (Figure 3). The selective sweep on CFA 26 was detected with allele-matching in English bulldog (CFA 26:6,785,609-11,282,297 bp; only 3 out of 368 SNPs with non-zero MAF) and French bulldog (CFA 26:8,548,679-9,227,043; 48 SNPs involved with MAF equal to zero) (Figure 3, Additional file 4). Although the ROH was not apparent in the Pug in Figure 3, in the segment of the ROH shared between Boxer and English and French bulldogs a good number of SNPs in Pug samples showed the same genotypes as the other three brachycephalic breeds. In addition, the SNPs comprised between 8,565,784 and 8,620,015 bp (~55 Kb) were nearly fixed in the Pug (Additional file 4). In contrast to the Boxer, a distribution of genotypes in HWE was seen for BICF2G630807104 (CFA 26:4,222,068 bp) in the bulldog breeds studied whereas it was fixed in the Pug (data not shown).

Within the SNPs making up the Illumina's CanineSNP20 only two covered the CNV on CFA 9 (Additional file 5), both of them highly monomorphic with the exception of the SNP at position 20,274,406 bp for the Shar pei samples for which an excess of heterozygous genotypes (HWE test p-value < 0.001) were observed. A pattern of excessive heterozygous genotypes was observed for the region corresponding to the CNV on CFA 9 in the breeds genotyped with the Illumina's CanineHD Beadchip (Additional file 6).

### Genetic content and functional annotation analysis

The region of decreased heterozygosity which was observed on CFA 1 in our study overlapped with a region previously associated with canine brachycephaly [7]. This was detected using dogs from brachycephalic breeds and non-brachycephalic breeds to perform across-breed association and selection mapping. Both strategies identified a region on CFA 1 at 59 Mb. The decrease in the averaged observed heterozygosity of brachycephalic dogs relative to non-brachycephalic dogs is indicative of a selective sweep at this position. Genes which have been associated with brachycephaly on CFA 1 include: *THBS2 *[Ensembl:ENSCAFG00000000874], which is expressed in bone and cartilage during development and in the adult skeleton [10], and *SMOC2 *[Ensembl:ENSCAFG00000000868], similar in sequence to *BM-40 *[Ensembl:ENSCAFG00000017855] which is expressed primarily during embryogenesis and in adult bone tissue [11].

**Figure 4:**
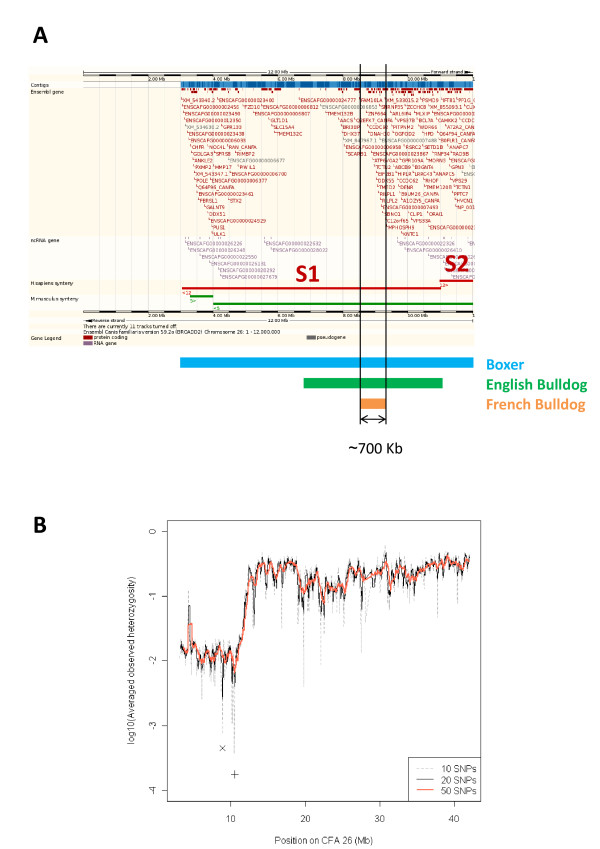
**Genetic content of the ROH on CFA 26**. **(a) **Annotated elements in the dog genome (CanFam2.0) as well as regions of synteny with *H. sapiens *and *M. musculus *are shown. CFA 26:3,008,718-11,914,284 bp maps to two syntenic regions in *H. sapiens*: Hs 12:121,547,433-133,784,108 bp (S1) and Hs 12:110,448,771-121,498,418 (S2). The end position for S2 is defined relative to the end of the ROH in dog though indeed synteny extends longer. Note that the order of the syntenic regions relative to the dog sequence indicates rearrangements events in the species; also, the different orientation of the sequences, indicated with arrows preceding the chromosome number in the compared species, designates an inversion in the orientation between *H. sapiens *and *C. familiaris *for S1. Regions of extended homozygosity for Boxer, English bulldog and French bulldog and the region of ~700 Kb shared in all three are indicated. **(b) **Averaged observed heterozygosity along CFA 26 in Boxer. Minimum values are shown for three window sizes: 10- and 50-SNPs, CFA 26:8.86 Mb (x); 20-SNPs, CFA 26:10.44 Mb (+).

The ROH in Boxer on CFA 26:3,008,718-11,914,284 bp contained 135 annotated elements of which 95 (71.9%) were genes with an associated name; this level was similar to that observed in the whole dog genome (69.7%). The ROH from CFA 26 mapped to two adjacent syntenic regions on *Homo sapiens *chromosome (Hs) 12 (Figure 4a). Advantage was taken of the fact that the region in the human genome syntenic to the region of interest on CFA 26 was better annotated and could be used to perform functional annotation analysis through the use of Ingenuity Pathways Analysis software [12]. One hundred and six dog to human orthologs annotated elements were used as input, resulting in a list of biological processes and disease categories that would be enriched given the genes in the region of interest. Amongst functional categories related to biological processes, skeletal and muscular system development and function as well as tissue morphology were the two most significantly associated categories (Additional file 7). Also, the selective sweep on CFA 26 contained genes that are linked to inherited diseases overrepresented in the Boxer [4] (Table 3). Lymphoblastic lymphoma is a type of non-Hodgkin lymphoma characterised by uncontrolled growth of either T- or B-cells and associated with *POLE *[Ensembl:ENSCAFG00000006215]. The frequency of T-cell lymphoma in the Boxer is higher than in other breeds [13-15]. Dilated cardiomyopathy (CMD) [OMIA:327] is a disorder characterised by cardiac enlargement (especially of the left ventricle), poor myocardial contractility, and congestive heart failure. *MYL2 *[Ensembl:ENSCAFG00000008524] is involved in the development of the sarcomere and muscle contraction and has also been associated with cardiomyopathy of the heart ventricle.

**Table 3:** Dog genes and human orthologs within the selective sweep on CFA 26.

*Boxer*					
**Human gene**	**Dog gene**	**Start (bp)**	**End (bp)**	**Ensembl Gene ID**	**Annotation**

*HIP1R*	*HIP1R*	9,641,249	9,654,192	ENSCAFG00000007764	Development of vertebral column
*P2RX4*	*Q64F94_CANFA*	10,917,230	10,933,173	ENSCAFG00000008363	Contraction (and relaxation) of cardiac muscle
*P2RX7*	*B0FLR1_CANFA*	10,959,759	11,002,514	ENSCAFG00000008401	Bone mineral density of skeleton Formation of perichondral bone Resorption of trabecular bone
*ATP2A2*	*AT2A2_CANFA*	11,167,290	11,232,956	ENSCAFG00000008434	Contraction (and relaxation) of cardiac muscle Contraction and relaxation of papillary muscle Elongation of cardiomyocytes
*POLE*	*POLE*	3,436,073	3,481,729	ENSCAFG00000006215	***Lymphoblastic lymphoma ***
*MYL2*	*NP_001003069.1*	11,482,695	11,650,673	ENSCAFG00000008524	Contraction of cardiac muscle Development of sarcomere ***Cardiomyopathy of heart ventricle***

*Boxer, English and French bulldogs*					

**Human gene**	**Dog gene**	**Start (bp)**	**End (bp)**	**Ensembl Gene ID**	**Annotation**

*CCDC92*	*CCDC92*	8,759,998	8,790,267	ENSCAFG00000006996	Amino Acid Metabolism Protein Synthesis Small Molecule Biochemistry
*ATP6V0A2*	*ATP6V0A2*	8,929,687	8,968,952	ENSCAFG00000007234	***Cutis laxa*** ***Wrinkly skin syndrome***
*EIF2B1*	*EIF2B1*	9,029,462	9,038,275	ENSCAFG00000007434	Gene Expression ***Leukoencephalopathy with vanishing white matter*** Protein Synthesis
*DDX55*	*DDX55*	9,040,108	9,053,306	ENSCAFG00000007452	***Cancer*** ***Infection Mechanism (by HIV)***
*TMED2*	*TMED2*	9,056,220	9,064,265	ENSCAFG00000007468	***Cancer*** ***Infection Mechanism (by HIV)***
*SETD8*		9,188,951	9,202,238	ENSCAFG00000007493	Tissue development and cell cycle

The upper part of the table (Boxer) refers to the selective sweep present in the Boxer only (CFA 26:3,008,718-11,914,284 bp). The lower part of the table (Boxer, English and French bulldogs) refers to the part of the selective sweep shared in the three breeds CFA 26:8,548,679-9,227,043 bp. Genomic positions in the dog genome and their annotated involvement in biological process and diseases (italic bold) are listed.

CFA 26:8,548,679-9,227,043 bp defined the region where extended homozygosity was common in Boxer, English bulldog and French bulldog (Figure 4a), which comprised six genes significant in the functional annotation analysis (Table 3). Of these genes, *ATP6V0A2 *[Ensembl:ENSCAFG00000007234] and *EIF2B1 *[Ensembl:ENSCAFG00000007434] are of special interest because they are involved in genetic disorders in humans. Various loss-of-function mutations of *ATP6V0A2 *[Ensembl:ENSCAFG00000007234], which encodes the alpha-2 subunit of the V-type H+ ATPase, resulting in impaired glycosilation of proteins during synthesis cause autosomal recessive cutis laxa (ARCL) type II [OMIM:219200] and some cases of wrinkly skin syndrome [OMIM:278250] [16]. Mutations in each of the five subunits of the translation initiation factor eIF2B, including eIF2a encoded by *EIF2B1 *[Ensembl:ENSCAFG00000007434], can cause leukoencephalopathy with vanishing white matter (VWM, [OMIM:603896]) [17]. VWM is a neurological disorder manifesting progressive cerebellar ataxia, spasticity, inconstant optic atrophy and relatively preserved mental abilities. *SETD8 *[Ensembl:ENSG00000183955] encodes a lysine methyltransferase that regulates tumor suppressor p53 protein [18]. To note, the region of ~55 Kb of nearly complete homozygosity in the Pug is both upstream of these six genes and within the region of extended homozygosity shared amongst the three breeds. Interestingly, (i) the genes involved in skeletal and muscular system development and tissue morphology that were significant in the functional annotation analysis in the Boxer only (with the exception of *POLE *[Ensembl:ENSCAFG00000006215]) and (ii) the region shared in Boxer and English and French bulldogs, are both located within the region on CFA 26 that showed the greatest decay in the averaged observed heterozygosity (Figure 4b).

The region of alternate heterozygous/homozygous genotypes patterns observed for CFA 9 in the Boxers overlapped perfectly to a CNV previously described as being polymorphic in a number of dog breeds [19,20]. This 1.5-Mb CNV region contained three protein coding genes, one of which had two reported transcript variants, and four non coding RNA genes (Additional file 8). Analysis of Gene Ontology Biological Process (GO BP) terms revealed that the two transcript variants of the protein coding gene *VPS13D *[Ensembl:ENSCAFG00000016397] (CFA 9:21,079,541-21,164,823 bp) were associated with processes of protein localization and viral envelope fusion with host membrane (GO:0008104 and GO:0019064, respectively). This gene was also mapped to the Hs 1:12,290,124-12,572,099 bp but only the protein localization term was associated (GO:0008104). The remaining annotated elements had neither GO BP terms associated nor homology in *H. sapiens*.

## Discussion

The substitution of a strongly selected mutation produces a selective sweep on the frequency of neutral alleles at linked loci characterised by a reduction of the local genetic variation [21-23]. Two selective sweeps detected on CFAs 1 and 26 in the Boxer genome were replicated in a larger sample size of the same breed obtained from a different geographical location and genotyped for a panel of SNPs of higher density. Assessing both the presence of these regions in other breeds and their genetic content can provide information on how they affect the phenotype and relate to the ancestral origin of breeds.

In our study, the selective sweep previously associated with brachycephaly on CFA 1 [7] was replicated in a larger sample of Boxers and in samples from other brachycephalic breeds. Moreover, samples in this study were from a different geographic area compared to the previous work [7] (Europe and US, respectively), suggesting the selective sweep is shared in the two populations within each breed.

The selective sweep on CFA 26 indicates strong artificial selection of a trait of interest in the Boxer, although the phenotypic trait resulting from this particular selective sweep is unknown. The sweep was not present in the Iberian wolf, one ancient breed (Shar pei), Labrador retrievers, German shepherd dogs or four hound breeds. On the other hand, it was present, although in shorter length, in English and French bulldogs, breeds that share with the Boxer the brachycephalic trait and a related breed creation process. Altogether, these results suggest that the selection of the sweep predated the formation of Boxer and both bulldog breeds. It is known that the English bulldog contributed to the breed creation of both Boxer and French bulldog breeds [24]. The Boxer is believed to have originated from a long-existing and now extinct German breed, the Bullenbeisser, which was crossed with a small number of English bulldog exemplars exported from the UK. Likewise, the French bulldog originated from toy varieties of English bulldog that were more popular in France. Moreover, it is interesting that the region of the selective sweep common in the three breeds coincides with the lowest reduction in the heterozygosity along the sequence (Figure 4). In selective sweeps, the reduction of genetic variation is lowest at the site of directional selection and not as great at distant sites due to recombination, although asymmetry in the valleys of reduced heterozygosity may provide imprecise information about the location of the sweep [25]. Based on our data, it is hard to assess whether the selective sweep on CFA 26 shared in Boxer and English and French bulldogs was also present in the Pug, also a brachycephalic breed, because only a short segment of reduced polymorphism within the sweep was observed in this breed (~55 Kb). Nonetheless, the history of the Pug differs from that of these three breeds mentioned before. The Pug dates to the ancient China and it is suggested that interbreeding with Pekingese, Japanese chin and possibly Shih tzu contributed to the breed creation process. Pugs were imported to Europe through Holland around 1,600s [24].

A possible scenario is that the standing neutral variation on CFA 26 present in the original English bulldog was passed to both Boxer and French bulldog during the breed creation process. Some variants would have been beneficial thereafter when selection of brachycephaly started, which is reasonable to think that happened during the breeds creation process since brachycephaly is a breed standard in these three types of dogs. Thus, strong selection of variants close to the position 8-10 Mb on CFA 26 contributing to brachycephaly might have swept nearby genetic variation. Variable selective sweep length in the three breeds would response to different breed histories as it depends on the strength of selection, the amount of recombination and the population size [21,22,25]. Therefore, if one assumes the recombination rate to be similar across breeds for a given chromosome region, different across-breeds strength of selection and population sizes might have probably caused the variable length in the sweeps on CFA 26, which in the Boxer is more than ten times larger than in the French bulldog.

Altogether we suggest that CFA 26 may contain a footprint of selection for brachycephaly, especially in the Boxer. A brachycephalic head with a distinctive broad and blunt muzzle is a unique phenotype of the Boxer and particular attention is given to this trait by the Boxer breeding community. Although brachycephaly has been mapped to CFA 1 and the greatest association was greater than 100 times more significant than the second highest, Bannasch *et al *[7] suggested that the complex nature of the brachycephalic head phenotype may be the result of associations across multiple chromosomes. Verification as to whether the genome-wide significant markers on CFA 26, the second highest association, previously reported [7] are within the ROH on CFA 26 in our data would provide some support for a link between this region and selection for brachycephaly. Genes on CFA 1 which have been associated with brachycephaly are involved in skeletal development [7,10,11]. Similarly, genes significant in functional annotation analysis of our data were associated with skeletal and muscular system development and function as well as tissue morphology biological process (Table 3).

It is possible that the selection for certain breed-specific loci or locus might be in linkage disequilibrium with detrimental variants at other genes. Interestingly, some of the genes in the selective sweep region on CFA 26 could be related to diseases that are reported to be more common in the Boxer breed, particularly cancer (lymphoblastic lymphoma) and cardiovascular disorders (CMD) [4].

In addition, we could observe in our data a previously reported CNV on CFA 9 polymorphic in multiple dog breeds [19,20], providing evidence for within-breed variation in the number of segment copies. Our data suggest that the CNV on CFA 9 is present and variable in the 273 Boxers used in this study (Figure 1b, 2), as well as in other breeds such as German shepherd dog, Pug and English and French bulldogs (Additional file 6). We suggest this CNV may be also possibly present in the Shar pei (Additional file 5) although in this breed it should be confirmed with a panel of higher SNP density. It might be that variable numbers of copies of a gene contained within the CNV such as *VPS13D *[Ensembl:ENSCAFG00000016397], which is involved in entrance of virus into the host cell, might be functional in the susceptibility to viral infection. Likewise, the non coding RNAs (ncRNAs) and small nuclear RNAs (snRNAs) which precede a region relatively rich in genes (data not shown) could be functional in regulatory processes.

## Conclusion

We have identified a selective sweep in excess of 8 Mb on CFA 26 in the Boxer which is not present in Iberian wolves or non-brachycephalic dog breeds. This region is a candidate for strong artificial selection in the Boxer for a trait of interest, possibly brachycephaly, and the inadvertent selection of genes during the enrichment for a certain phenotype may have given rise to an increased incidence of certain related afflictions in the breed. The fact that the selective sweep is also present in English and French bulldogs provides genetic evidence of a shared history of the three breeds.

Furthermore, we provide supporting evidence for two previously described regions: a selective sweep on CFA 1 associated with canine brachycephaly and a CNV on CFA 9 which is polymorphic in multiple dog breeds and contains genetic elements with potential biological implications.

## Methods

### Sample collection

A set of 27 Boxer samples from the UK (denoted as set A) were collected as residual samples from dogs taken for clinical investigation. They were selected from a large archive of DNA samples (UK Companion Animal DNA Archive, University of Manchester) and all samples had informed owner consent. A second set of 274 Boxer samples were collected from Spain, Greece, Italy and Portugal (denoted as set B); samples from Spain represented > 90% of set B. Dogs in this set and the remaining breeds in Table 1 came from the Hospital Clínic Veterinari of the Universitat Autònoma de Barcelona, veterinary clinics or dog owners.

### DNA extraction and genotyping

DNA was extracted from peripheral blood or bone marrow samples using either QIAamp^® ^DNA Blood Mini Kit (QIAGEN) or PureLink™ Genomic DNA (Invitrogen). Set A was genotyped at 22,362 SNPs with Illumina's CanineSNP20 BeadChip at The Genome Centre, Queen Mary University of London, UK. Set B and German shepherd dog samples were genotyped at 174,376 markers using Illumina's CanineHD BeadChip at The Centre National de Génotypage, France. The remaining samples were genotyped as indicated in Table 1 at the Universitat Autònoma de Barcelona, Spain.

### Data cleaning

Data cleaning was conducted using PLINK and R packages [26,27]. Set A was filtered to have individual and marker call rates > 90%, resulting in 25 Boxers and 22,300 SNPs left for analysis. The same filters were applied to set B and, moreover, in this set we also excluded intensity probes, markers on the boundary autosomal region on chromosome CFA X as well as those SNPs on the non-pseudoautosomal region on CFA X for which heterozygous genotypes in male samples were observed. All samples in set B had an individual call rate > 90% but one sample was excluded as it appeared as an outlier when the first two dimensions of the multidimensional scaling analysis were plotted (Additional file 9). This resulted in 273 individuals with 171,772 SNPs each left for analysis.

### Statistical analysis

Averaged observed heterozygosity was calculated as the moving average of the observed heterozygosity using 50-SNPs windows both for set A (20,451 windows) and set B (169,812 windows). In each set the 1% of windows with the lowest averaged observed heterozygosity was selected (Additional file 2); windows spaced less than fifty times the mean SNP density (bp/SNP) of the beadchip used were considered as single regions of homozygosity. ROHs common in both sets were defined as those overlapping in at least one SNP. The analysis was also performed on the dataset with the SNPs with Hardy-Weinberg Equilibrium test p-value > 0.005 and the identified ROHs presented in Table 2 correspond to this second analysis.

### Genetic content and functional annotation analysis

The position of the CNV detected on CFA 9 was defined as the union resulting from our data and the positions annotated in the Ensembl database [28] in two previous works describing this CNV [19,20]. This resulted in a region at CFA 9:19,778,695-21,332,928 bp that was searched for Gene Ontology biological process (GO BP) terms using Biomart [29] and regions of synteny with *H. sapiens*. For the ROH on CFA 26 Ensembl IDs of the annotated elements in the syntenic region at Hs 12:108,311,620-133,784,108 bp were retrieved using Biomart [29] and used as input for functional annotation analysis. The annotated genes in Hs 12:108,311,620-133,784,108 bp were tested for enrichment of certain biological functions or diseases by comparison with the annotations from the Ingenuity database for mouse, rat and human genomes [12]. Right-tailed Fisher's exact test was used to calculate a p-value determining the probability that each biological function and/or disease assigned to that data set was due to chance alone. The categories of diseases associated with the region of interest were compared with the reported inherited diseases in the Boxer breed [4].

## Authors' contributions

AS, ASB, LA, LK, OF and WO designed the experiment. ASB, LA, OF and WO supervised the project and gave conceptual advice. AS, JQO, LK, and VMD collected the samples. JQO and VMD performed DNA extraction and genotyping. AS provided technical support for the data analysis. JQO performed, data cleaning, statistical and genetic content analysis and wrote the manuscript. AS, LA, OF, VMD and WO edited the manuscript. All authors read and approved the final manuscript.

## Supplementary Material

Additional file 1 **Comparison of the ROHs on CFA 1, 10 and 26 common in sets A **(Figure S1a-e**) and B (**Figure S1f-j**)**. Red lines indicate ROH as defined in each set. Click here for file

Additional file 2 **Summary statistics of the 50-SNP sliding windows**. Click here for file

Additional file 3 **Comparison of ROHs identified in Set B and Set B pruned for SNPs significantly deviating from HWE**. Note that for the region setB_06 two different regions were detected and that the end of setB_07 overlaps with start of setB_08. Click here for file

Additional file 4 **Genotypes of samples from brachycephalic breeds for the segment of the ROH on CFA 26 shared between Boxer and English and French bulldogs**. For the Boxer, a random sample of 20 dogs is displayed. Click here for file

Additional file 5 **Genotypes for the CNV on CFA 9 in samples from other breeds than Boxer and Iberian wolf genotyped with the Illumina's CanineSNP20**. Chromosome positions corresponding to the described CNV are highlighted in green and genotypes grouped by breed. Homozygous genotypes are coded as '11' (grey) and '22' (orange), respectively, and heterozygous genotypes as '12' (blue); '00' represents missing values (white). Click here for file

Additional file 6 **Genotypes for the CNV on CFA 9 in samples from other breeds than Boxer and Iberian wolf genotyped with the Illumina's CanineHD**. Chromosome positions corresponding to the described CNV are highlighted in green and genotypes grouped by breed. Homozygous genotypes are coded as '11' (grey) and '22' (orange), respectively, and heterozygous genotypes as '12' (blue); '00' represents missing values (white). Click here for file

Additional file 7 **FAA_long: functional annotation categories are classified either as biological processes (BP) or diseases (DIS)**. Function annotations that either involve genes laying in the region of homozygosity common in Boxer, English and French bulldogs (orange) or relate to inherited diseases overrepresented in the Boxer (blue) are highlighted. FAA_GenomicPosition: annotated elements in the selective sweep on chromosome 26 and in the syntenic region in the human genome. Whether annotated elements lay in the region of homozygosity in each breed (Boxer and English and French bulldogs) is indicated. Annotated elements significant in the functional annotation analysis are highlighted (orange). InheritedDiseases: inherited diseases reported in Boxer, English and French bulldogs [4]. Of these, those significant in the functional annotation analysis are highlighted (blue). Click here for file

Additional file 8 **File containing genetic content, Gene Ontology Biological Process (GO BP) annotation and synteny information for the CNV on CFA 9**. Click here for file

Additional file 9 **Multidimensional scaling (MDS) plot of the two first dimensions C1 and C2**. The excluded outlier samples are indicated by the arrows. Click here for file
