# Supplementary figures and images for: A selective sweep of >8 Mb on chromosome 26 in the Boxer genome
Source: BMC Genomics. 2011 Jul 1;12:339. doi: 10.1186/1471-2164-12-339 (PMC3152542; doi:10.1186/1471-2164-12-339)

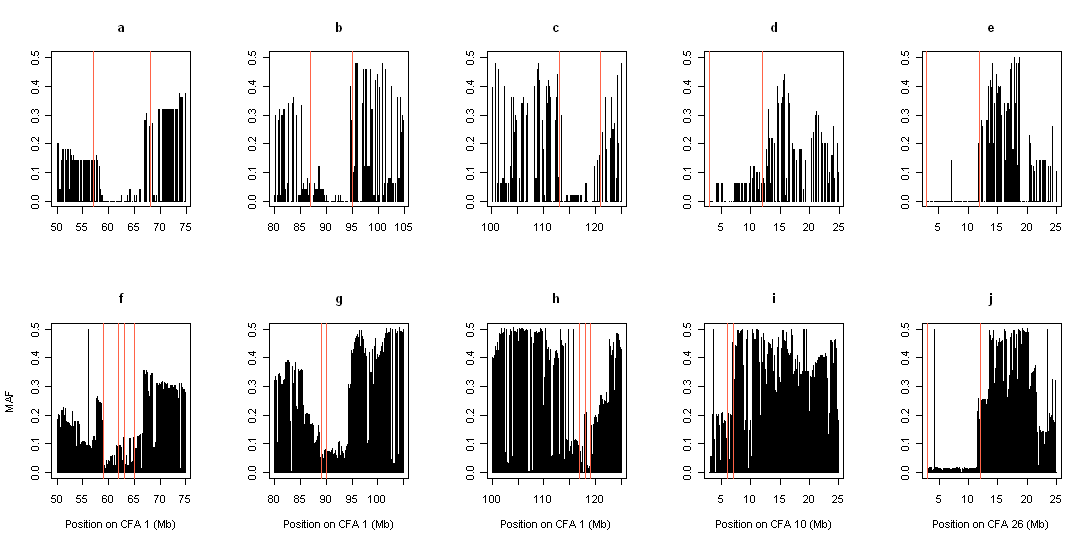

Supplement: Additional file 1 — Comparison of the ROHs on CFA 1, 10 and 26 common in sets A (Figure S1a-e) and B (Figure S1f-j). Red lines indicate ROH as defined in each set. [file 1471-2164-12-339-S1.PNG]

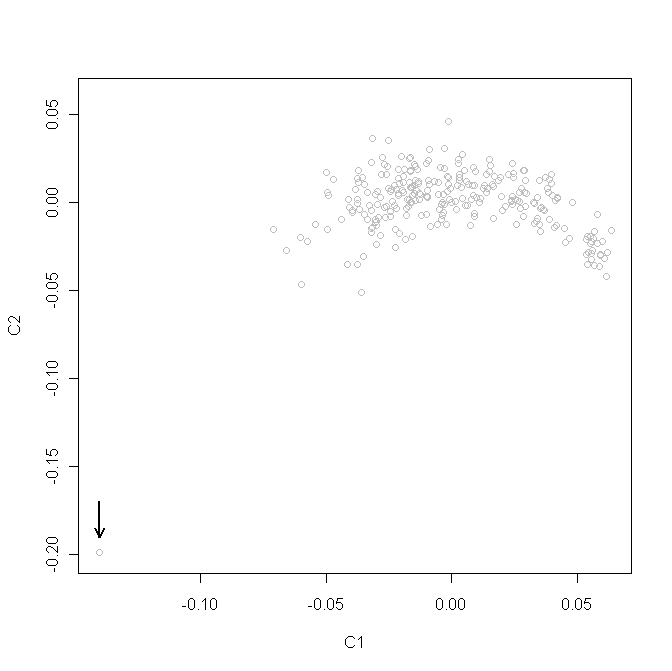

Supplement: Additional file 9 — Multidimensional scaling (MDS) plot of the two first dimensions C1 and C2. The excluded outlier samples are indicated by the arrows. [file 1471-2164-12-339-S9.PNG]
