# Supplementary material for: A selective sweep of >8 Mb on chromosome 26 in the Boxer genome
Source: BMC Genomics. 2011 Jul 1;12:339. doi: 10.1186/1471-2164-12-339 (PMC3152542; doi:10.1186/1471-2164-12-339)
Supplement: Additional file 2 — Summary statistics of the 50-SNP sliding windows. [file 1471-2164-12-339-S2.DOC]

|  | **Set A** | **Set B** | **Set B (HWE test p-value > 0.005)** |
| --- | --- | --- | --- |
| Number of SNPs | 22,362 | 171,772 | 168,239 |
| Number of windows | 20,451 | 169,812 | 166,279 |
| Normalized heterozygosity  1.0% quantile  0.1% quantile | 0.051  0.006 | 0.046  0.011 | 0.045  0.011 |
| Mean SNP density (Kb/SNP) | 102 | 14 | 14 |
| Maximum spacing between SNPs (Kb) | 5,552 | 3,382 | 3,382 |
