# Supplementary material for: A selective sweep of >8 Mb on chromosome 26 in the Boxer genome
Source: BMC Genomics. 2011 Jul 1;12:339. doi: 10.1186/1471-2164-12-339 (PMC3152542; doi:10.1186/1471-2164-12-339)
Supplement: Additional file 3 — Comparison of ROHs identified in Set B and Set B pruned for SNPs significantly deviating from HWE. Note that for the region setB_06 two different regions were detected and that the end of setB_07 overlaps with start of setB_08. [file 1471-2164-12-339-S3.DOC]

|  |  | ***Set B*** |  | | ***Set B***  ***(HWE test p-value > 0.005)*** |  |  |  |
| --- | --- | --- | --- | --- | --- | --- | --- | --- |
| **Region ID** | **CFA** | **BP1** | | **BP2** | **BP1** | **BP2** | **Difference in BP1** | **Difference in BP2** |
| SetB_01 | 1 | 26,672,978 | | 27,730,188 | 26,672,978 | 27,730,188 | 0 | 0 |
| SetB_02 | 1 | 45,207,395 | | 46,286,798 | 45,218,029 | 46,286,798 | 10,634 | 0 |
| SetB_03 | 1 | 58,710,420 | | 61,801,815 | 58,732,954 | 61,801,815 | 22,534 | 0 |
| SetB_04 | 1 | 62,722,220 | | 65,201,836 | 62,722,220 | 65,190,321 | 0 | 11,515 |
| SetB_05 | 1 | 89,051,381 | | 90,230,941 | 89,187,131 | 90,230,941 | 135,750 | 0 |
| SetB_06 | 1 | 91,184,730 | | 92,038,243 | 102,454,189 | 103,320,473 | - | - |
| SetB_07 | 1 | 116,683,627 | | 118,107,497 | 116,688,554 | 118,107,497 | 4,927 | 0 |
| SetB_08 | 1 | 117,979,514 | | 118,963,939 | 117,979,514 | 118,963,939 | 0 | 0 |
| SetB_09 | 2 | 22,715,411 | | 23,895,662 | 22,715,411 | 24,024,566 | 0 | 128,904 |
| SetB_10 | 3 | 3,030,299 | | 3,903,071 | 3,030,299 | 3,903,071 | 0 | 0 |
| SetB_11 | 5 | 4,697,408 | | 6,247,457 | 4,697,408 | 6,247,457 | 0 | 0 |
| SetB_12 | 6 | 25,815,666 | | 26,601,998 | 25,815,666 | 26,601,998 | 0 | 0 |
| SetB_13 | 6 | 42,437,711 | | 43,447,406 | 42,437,711 | 43,955,158 | 0 | 507,752 |
| SetB_14 | 6 | 58,478,112 | | 59,356,441 | 58,580,737 | 59,356,441 | 102,625 | 0 |
| SetB_15 | 9 | 3,529,583 | | 4,363,516 | 3,529,583 | 4,304,182 | 0 | 59,334 |
| SetB_16 | 10 | 5,626,769 | | 6,840,140 | 5,626,769 | 6,702,961 | 0 | 137,179 |
| SetB_17 | 10 | 59,180,359 | | 60,456,532 | 59,180,359 | 60,456,532 | 0 | 0 |
| SetB_18 | 10 | 65,161,142 | | 66,656,760 | 65,209,901 | 66,606,742 | 48,759 | 50,018 |
| SetB_19 | 10 | 68,323,860 | | 69,026,432 | 68,323,860 | 69,026,432 | 0 | 0 |
| SetB_20 | 13 | 39,894,733 | | 40,797,838 | 39,705,171 | 40,797,838 | 189,562 | 0 |
| SetB_21 | 14 | 19,789,648 | | 20,470,904 | 19,806,989 | 20,470,904 | 17,341 | 0 |
| SetB_22 | 18 | 6,868,787 | | 7,908,159 | 6,868,787 | 7,908,159 | 0 | 0 |
| SetB_23 | 20 | 7,816,139 | | 8,638,174 | 7,816,139 | 8,635,017 | 0 | 3,157 |
| SetB_24 | 24 | 24,444,170 | | 27,387,620 | 24,444,170 | 27,387,620 | 0 | 0 |
| SetB_25 | 24 | 28,845,341 | | 29,848,829 | 28,845,341 | 29,848,829 | 0 | 0 |
| SetB_26 | 26 | 3,008,718 | | 11,914,284 | 3,008,718 | 11,914,284 | 0 | 0 |
| SetB_27 | 30 | 38,126,268 | | 38,689,821 | 38,126,268 | 38,689,821 | 0 | 0 |
