# Supplementary material for: A selective sweep of >8 Mb on chromosome 26 in the Boxer genome
Source: BMC Genomics. 2011 Jul 1;12:339. doi: 10.1186/1471-2164-12-339 (PMC3152542; doi:10.1186/1471-2164-12-339)
Supplement: Additional file 4 — Genotypes of samples from brachycephalic breeds for the segment of the ROH on CFA 26 shared between Boxer and English and French bulldogs. For the Boxer, a random sample of 20 dogs is displayed. [file 1471-2164-12-339-S4.PDF]

Position at CFA 26 (bp)

Breed	8,548,679	8,565,784	8,573,105	8,589,815	8,591,289	8,607,815	8,610,786	8,620,015	8,635,745	8,651,371	8,694,743	8,724,281	8,746,093	8,790,483	8,797,934	8,813,638	8,826,462	8,840,367	8,850,907	8,857,340	8,897,177	8,904,407	8,913,141	8,915,745
Boxer	GG	TT	AA	AA	GG	TT	TT	GG	TT	CC	GG	AA	AA	TT	GG	AA	AA	CC	AA	AA	AA	GG	CC	GG
	GG	TT	AA	AA	GG	TT	TT	GG	TT	CC	GG	AA	AA	TT	GG	AA	AA	CC	AA	AA	AA	GG	CC	GG
	GG	TT	AA	AA	GG	TT	TT	GG	TT	CC	GG	AA	AA	TT	GG	AA	AA	CC	AA	AA	AA	GG	CC	GG
	GG	TT	AA	AA	GG	TT	TT	GG	TT	CC	GG	AA	AA	TT	GG	AA	AA	CC	AA	AA	AA	GG	CC	GG
	GG	TT	AA	AA	GG	TT	TT	GG	TT	CC	GG	AA	AA	TT	GG	AA	AA	CC	AA	AA	AA	GG	CC	GG
	GG	TT	AA	AA	GG	TT	TT	GG	TT	CC	GG	AA	AA	TT	GG	AA	AA	CC	AA	AA	AA	GG	CC	GG
	GG	TT	AA	AA	GG	TT	TT	GG	TT	CC	GG	AA	AA	TT	GG	AA	AA	CC	AA	AA	AA	GG	CC	GG
	GG	TT	AA	AA	GG	TT	TT	GG	TT	CC	GG	AA	AA	TT	GG	AA	AA	CC	AA	AA	AA	GG	CC	GG
	GG	TT	AA	AA	GG	TT	TT	GG	TT	CC	GG	AA	AA	TT	GG	AA	AA	CC	AA	AA	AA	GG	CC	GG
	GG	TT	AA	AA	GG	TT	TT	GG	TT	CC	GG	AA	AA	TT	GG	AA	AA	CC	AA	AA	AA	GG	CC	GG
	GG	TT	AA	AA	GG	TT	TT	GG	TT	CC	GG	AA	AA	TT	GG	AA	AA	CC	AA	AA	AA	GG	CC	GG
	GG	TT	AA	AA	GG	TT	TT	GG	TT	CC	GG	AA	AA	TT	GG	AA	AA	CC	AA	AA	AA	GG	CC	GG
	GG	TT	AA	AA	GG	TT	TT	GG	TT	CC	GG	AA	AA	TT	GG	AA	AA	CC	AA	AA	AA	GG	CC	GG
	GG	TT	AA	AA	GG	TT	TT	GG	TT	CC	GG	AA	AA	TT	GG	AA	AA	CC	AA	AA	AA	GG	CC	GG
	GG	TT	AA	AA	GG	TT	TT	GG	TT	CC	GG	AA	AA	TT	GG	AA	AA	CC	AA	AA	AA	GG	CC	GG
	GG	TT	AA	AA	GG	TT	TT	GG	TT	CC	GG	AA	AA	TT	GG	AA	AA	CC	AA	AA	AA	GG	CC	GG
	GG	TT	AA	AA	GG	TT	TT	GG	TT	CC	GG	AA	AA	TT	GG	AA	AA	CC	AA	AA	AA	GG	CC	GG
	GG	TT	AA	AA	GG	TT	TT	GG	TT	CC	GG	AA	AA	TT	GG	AA	AA	CC	AA	AA	AA	GG	CC	GG
	GG	TT	AA	AA	GG	TT	TT	GG	TT	CC	GG	AA	AA	TT	GG	AA	AA	CC	AA	AA	AA	GG	CC	GG
	GG	TT	AA	AA	GG	TT	TT	GG	TT	CC	GG	AA	AA	TT	GG	AA	AA	CC	AA	AA	AA	GG	CC	GG
English bulldog	GG	TT	AA	AA	GG	TT	TT	GG	TT	CC	GG	AA	AA	TT	GG	AA	AA	CC	AA	AA	AA	GG	CC	GG
	GG	TT	AA	AA	GG	TT	TT	GG	TT	CC	GG	AA	AA	TT	GG	AA	AA	CC	AA	AA	AA	GG	CC	GG
	GG	TT	AA	AA	GG	TT	TT	GG	TT	CC	GG	AA	AA	TT	GG	AA	AA	CC	AA	AA	AA	GG	CC	GG
	GG	TT	AA	AA	GG	TT	TT	GG	TT	CC	GG	AA	AA	TT	GG	AA	AA	CC	AA	AA	AA	GG	CC	GG
French bulldog	GG	TT	AA	AA	GG	TT	TT	GG	TT	CC	GG	AA	AA	TT	GG	AA	AA	CC	AA	AA	AA	GG	CC	GG
	GG	TT	AA	AA	GG	TT	TT	GG	TT	CC	GG	AA	AA	TT	GG	AA	AA	CC	AA	AA	AA	GG	CC	GG
	GG	TT	AA	AA	GG	TT	TT	GG	TT	CC	GG	AA	AA	TT	GG	AA	AA	CC	AA	AA	AA	GG	CC	GG
	GG	TT	AA	AA	GG	TT	TT	GG	TT	CC	GG	AA	AA	TT	GG	AA	AA	CC	AA	AA	AA	GG	CC	GG
	GG	TT	AA	AA	GG	TT	TT	GG	TT	CC	GG	AA	AA	TT	GG	AA	AA	CC	AA	AA	AA	GG	CC	GG
	GG	TT	AA	AA	GG	TT	TT	GG	TT	CC	GG	AA	AA	TT	GG	AA	AA	CC	AA	AA	AA	GG	CC	GG
Pug	GG	TT	AA	AA	GG	TT	TT	GG	TT	CC	GG	AA	AA	TT	GG	AA	AA	CC	AA	AA	AA	GG	CC	GG
	AG	TT	AG	AA	GG	TT	TT	GG	GT	CC	GG	AA	AA	TT	GG	AA	AA	CC	AA	AA	AA	GG	CC	GG
	AA	TT	AG	AA	GG	TT	TT	GG	TT	CC	GG	AA	AA	TT	GG	AG	AG	CC	AA	AG	AG	GG	CT	GT
	GG	TT	AA	AA	GG	TT	TT	GG	TT	CC	GG	AA	AA	TT	GG	AA	AA	CC	AA	AA	AA	GG	CC	GG
	GG	TT	AA	AA	GG	TT	TT	GG	TT	CC	GT	AA	AA	TT	GG	AA	AA	CC	AA	AA	AA	GG	CC	GT
	GG	TT	AA	AA	GG	TT	TT	GG	GG	CC	GG	AA	AA	TT	GG	AA	AA	CC	AA	AA	AA	GG	CC	GG
	AA	TT	AA	AA	GG	TT	TT	GG	TT	CC	GG	AA	AA	TT	GG	GG	GG	CC	AA	GG	GG	GG	TT	TT
	AG	TT	AA	AA	GG	TT	TT	GG	GT	CC	GG	AA	AA	TT	GG	AG	AG	CC	AA	AG	AG	GG	CT	GT
	GG	TT	AA	AA	GG	TT	TT	GG	GG	CC	GG	AA	AA	TT	GG	AA	AA	CC	AA	AA	AA	GG	CC	GG
	AG	TT	AG	AA	GG	TT	TT	GG	GT	CC	GG	AA	AA	TT	GG	AA	AA	CC	AA	AA	AA	GG	CC	GG

Breed	8,932,624	8,940,353	8,947,103	8,959,614	8,980,009	8,986,952	8,997,286	9,007,676	9,017,408	9,034,625	9,041,601	9,054,732	9,082,989	9,091,220	9,124,485	9,138,427	9,149,220	9,154,555	9,176,011	9,181,916	9,189,222	9,206,221	9,212,888	9,227,043
Boxer	GG	TT	AA	CC	TT	GG	TT	AA	AA	TT	CC	CC	TT	TT	GG	CC	CC	GG	GG	AA	AA	AA	CC	GG
	GG	TT	AA	CC	TT	GG	TT	AA	AA	TT	CC	CC	TT	TT	GG	CC	CC	GG	GG	AA	AA	AA	CC	GG
	GG	TT	AA	CC	TT	GG	TT	AA	AA	TT	CC	CC	TT	TT	GG	CC	CC	GG	GG	AA	AA	AA	CC	GG
	GG	TT	AA	CC	TT	GG	TT	AA	AA	TT	CC	CC	TT	TT	GG	CC	CC	GG	GG	AA	AA	AA	CC	GG
	GG	TT	AA	CC	TT	GG	TT	AA	AA	TT	CC	CC	TT	TT	GG	CC	CC	GG	GG	AA	AA	AA	CC	GG
	GG	TT	AA	CC	TT	GG	TT	AA	AA	TT	CC	CC	TT	TT	GG	CC	CC	GG	GG	AA	AA	AA	CC	GG
	GG	TT	AA	CC	TT	GG	TT	AA	AA	TT	CC	CC	TT	TT	GG	CC	CC	GG	GG	AA	AA	AA	CC	GG
	GG	TT	AA	CC	TT	GG	TT	AA	AA	TT	CC	CC	TT	TT	GG	CC	CC	GG	GG	AA	AA	AA	CC	GG
	GG	TT	AA	CC	TT	GG	TT	AA	AA	TT	CC	CC	TT	TT	GG	CC	CC	GG	GG	AA	AA	AA	CC	GG
	GG	TT	AA	CC	TT	GG	TT	AA	AA	TT	CC	CC	TT	TT	GG	CC	CC	GG	GG	AA	AA	AA	CC	GG
	GG	TT	AA	CC	TT	GG	TT	AA	AA	TT	CC	CC	TT	TT	GG	CC	CC	GG	GG	AA	AA	AA	CC	GG
	GG	TT	AA	CC	TT	GG	TT	AA	AA	TT	CC	CC	TT	TT	GG	CC	CC	GG	GG	AA	AA	AA	CC	GG
	GG	TT	AA	CC	TT	GG	TT	AA	AA	TT	CC	CC	TT	TT	GG	CC	CC	GG	GG	AA	AA	AA	CC	GG
	GG	TT	AA	CC	TT	GG	TT	AA	AA	TT	CC	CC	TT	TT	GG	CC	CC	GG	GG	AA	AA	AA	CC	GG
	GG	TT	AA	CC	TT	GG	TT	AA	AA	TT	CC	CC	TT	TT	GG	CC	CC	GG	GG	AA	AA	AA	CC	GG
	GG	TT	AA	CC	TT	GG	TT	AA	AA	TT	CC	CC	TT	TT	GG	CC	CC	GG	GG	AA	AA	AA	CC	GG
	GG	TT	AA	CC	TT	GG	TT	AA	AA	TT	CC	CC	TT	TT	GG	CC	CC	GG	GG	AA	AA	AA	CC	GG
	GG	TT	AA	CC	TT	GG	TT	AA	AA	TT	CC	CC	TT	TT	GG	CC	CC	GG	GG	AA	AA	AA	CC	GG
	GG	TT	AA	CC	TT	GG	TT	AA	AA	TT	CC	CC	TT	TT	GG	CC	CC	GG	GG	AA	AA	AA	CC	GG
	GG	TT	AA	CC	TT	GG	TT	AA	AA	TT	CC	CC	TT	TT	GG	CC	CC	GG	GG	AA	AA	AA	CC	GG
English bulldog	GG	TT	AA	CC	TT	GG	TT	AA	AA	TT	CC	CC	TT	TT	GG	CC	CC	GG	GG	AA	AA	AA	CC	GG
	GG	TT	AA	CC	TT	GG	TT	AA	AA	TT	CC	CC	TT	TT	GG	CC	CC	GG	GG	AA	AA	AA	CC	GG
	GG	TT	AA	CC	TT	GG	TT	AA	AA	TT	CC	CC	TT	TT	GG	CC	CC	GG	GG	AA	AA	AA	CC	GG
	GG	TT	AA	CC	TT	GG	TT	AA	AA	TT	CC	CC	TT	TT	GG	CC	CC	GG	GG	AA	AA	AA	CC	GG
French bulldog	GG	TT	AA	CC	TT	GG	TT	AA	AA	TT	CC	CC	TT	TT	GG	CC	CC	GG	GG	AA	AA	AA	CC	GG
	GG	TT	AA	CC	TT	GG	TT	AA	AA	TT	CC	CC	TT	TT	GG	CC	CC	GG	GG	AA	AA	AA	CC	GG
	GG	TT	AA	CC	TT	GG	TT	AA	AA	TT	CC	CC	TT	TT	GG	CC	CC	GG	GG	AA	AA	AA	CC	GG
	GG	TT	AA	CC	TT	GG	TT	AA	AA	TT	CC	CC	TT	TT	GG	CC	CC	GG	GG	AA	AA	AA	CC	GG
	GG	TT	AA	CC	TT	GG	TT	AA	AA	TT	CC	CC	TT	TT	GG	CC	CC	GG	GG	AA	AA	AA	CC	GG
	GG	TT	AA	CC	TT	GG	TT	AA	AA	TT	CC	CC	TT	TT	GG	CC	CC	GG	GG	AA	AA	AA	CC	GG
Pug	GG	TT	AA	CC	TT	GG	TT	AA	AA	TT	CC	CC	TT	TT	GG	CC	CC	GG	GG	AA	AA	AA	CC	GG
	AG	CT	AG	CT	CT	AG	CT	AG	AA	TT	AC	CT	AA	CC	AA	CC	CC	GG	AG	AC	AG	AT	CC	GT
	AG	CT	AG	CT	TT	GG	TT	AA	AA	TT	AA	CC	AA	CC	AA	CC	CC	GG	AA	CC	GG	TT	CC	GT
	GG	TT	AA	CC	TT	GG	TT	AA	AA	TT	CC	CC	TT	TT	GG	CC	CC	GG	GG	AA	AA	AA	CC	GG
	GG	TT	AA	CC	TT	GG	TT	AA	AA	TT	CC	CC	TT	TT	GG	CC	CG	AG	AG	AC	AG	AT	CC	GT
	AA	CC	GG	TT	CC	AA	CC	GG	AA	TT	CC	TT	AA	CC	AA	CC	CC	GG	GG	AA	AA	AA	CC	GG
	AA	CC	GG	TT	TT	GG	TT	AA	AA	TT	AA	CC	AA	CC	AA	CC	CC	GG	AA	CC	GG	TT	CC	GG
	AA	CC	GG	TT	CT	AG	CT	AG	AA	TT	AC	CT	AA	CC	AA	CC	CC	GG	AG	AC	AG	AT	CC	GG
	AA	CC	GG	TT	CC	AA	CC	GG	AA	TT	CC	TT	AA	CC	AA	CC	CC	GG	GG	AA	AA	AA	CC	GG
	AG	CT	AG	CT	CT	AG	CT	AG	AA	TT	AC	CT	AA	CC	AA	CC	CC	GG	AG	AC	AG	AT	CC	GT
