# Supplementary material for: A selective sweep of >8 Mb on chromosome 26 in the Boxer genome
Source: BMC Genomics. 2011 Jul 1;12:339. doi: 10.1186/1471-2164-12-339 (PMC3152542; doi:10.1186/1471-2164-12-339)
Supplement: Additional file 6 — Genotypes for the CNV on CFA 9 in samples from other breeds than Boxer and Iberian wolf genotyped with the Illumina's CanineHD. Chromosome positions corresponding to the described CNV are highlighted in green and genotypes grouped by breed. Homozygous genotypes are coded as '11' (grey) and '22' (orange), respectively, and heterozygous genotypes as '12' (blue); '00' represents missing values (white). [file 1471-2164-12-339-S6.PDF]

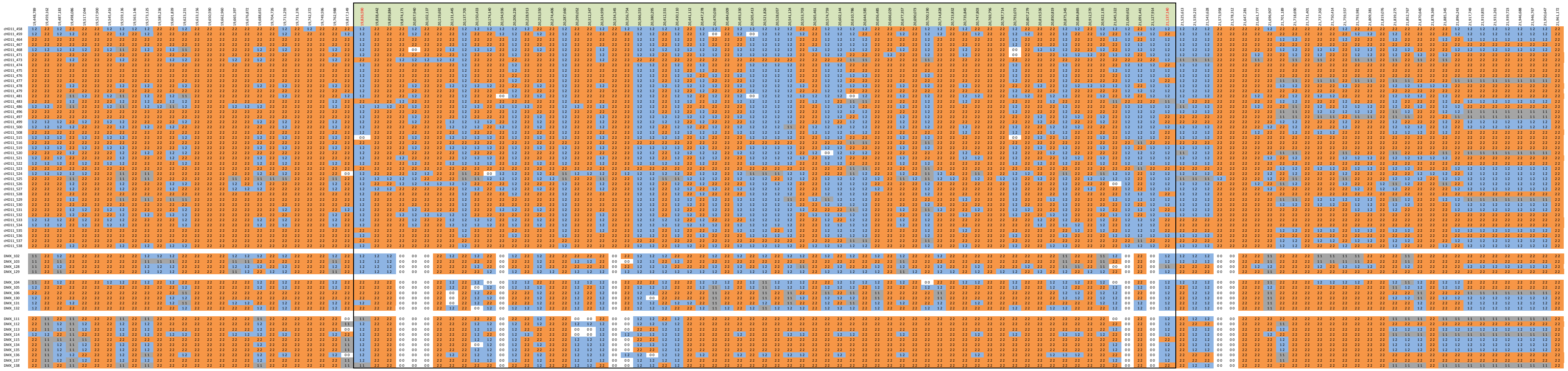
GSD
E. Bulldog
F. Bulldog
Pug
