# Supplementary material for: A selective sweep of >8 Mb on chromosome 26 in the Boxer genome
Source: BMC Genomics. 2011 Jul 1;12:339. doi: 10.1186/1471-2164-12-339 (PMC3152542; doi:10.1186/1471-2164-12-339)
Supplement: Additional file 5 — Genotypes for the CNV on CFA 9 in samples from other breeds than Boxer and Iberian wolf genotyped with the Illumina's CanineSNP20. Chromosome positions corresponding to the described CNV are highlighted in green and genotypes grouped by breed. Homozygous genotypes are coded as '11' (grey) and '22' (orange), respectively, and heterozygous genotypes as '12' (blue); '00' represents missing values (white). [file 1471-2164-12-339-S5.PDF]

	19,084,362	19,228,271	19,289,821	19,367,120	19,517,044	19,559,136	19,688,653	19,762,988	19,859,884	20,274,406	21,578,312	
LUP0563	1 1	2 2	1 1	1 1	2 2	2 2	1 1	1 1	2 2	2 2	2 2	**CIR**
LUP0593	1 1	2 2	1 1	1 2	2 2	2 2	1 2	1 1	2 2	2 2	2 2	
LUP0598	1 1	2 2	1 2	1 1	1 2	2 2	1 2	1 2	2 2	2 2	2 2	
LUP0553	1 2	1 2	1 2	1 2	1 2	2 2	2 2	1 1	2 2	2 2	2 2	
LUP0561	1 2	2 2	1 1	1 1	2 2	2 2	1 2	1 1	2 2	2 2	2 2	
LUP0566	1 2	2 2	1 2	1 2	2 2	2 2	1 2	1 2	2 2	2 2	2 2	
LUP0572	1 2	1 2	1 1	1 2	2 2	2 2	1 2	1 1	2 2	2 2	2 2	
LUP0577	1 2	1 2	2 2	2 2	2 2	1 2	1 2	2 2	1 2	2 2	2 2	
LUP0594	1 2	1 2	1 1	1 2	2 2	2 2	1 2	1 1	2 2	2 2	2 2	
LUP0595	1 2	2 2	1 2	1 2	2 2	2 2	1 1	1 2	2 2	2 2	2 2	
LUP0551	2 2	1 1	1 1	2 2	2 2	2 2	2 2	1 1	2 2	2 2	2 2	
LUP0574	2 2	1 1	1 2	1 2	2 2	2 2	1 2	1 2	2 2	2 2	2 2	
LUP0663_2	1 2	1 2	2 2	2 2	2 2	2 2	1 2	2 2	2 2	2 2	2 2	**CWH**
LUP0664_2	1 2	1 2	2 2	2 2	2 2	2 2	1 2	2 2	2 2	2 2	2 2	
LUP0666_2	1 2	1 2	2 2	1 2	2 2	2 2	2 2	2 2	1 2	2 2	2 2	
LUP0815	1 2	1 2	2 2	2 2	2 2	2 2	2 2	2 2	2 2	2 2	2 2	
LUP0820	1 2	1 2	2 2	2 2	2 2	2 2	2 2	2 2	2 2	2 2	2 2	
LUP0821	1 2	1 2	1 2	1 2	2 2	2 2	1 2	2 2	2 2	2 2	2 2	
LUP0823	1 2	1 2	1 2	1 1	2 2	2 2	0 0	2 2	1 2	2 2	2 2	
LUP0662	2 2	2 2	1 2	1 1	2 2	2 2	1 2	2 2	2 2	1 2	2 2	
LUP0665_1	2 2	2 2	2 2	1 2	2 2	2 2	2 2	2 2	2 2	2 2	2 2	
LUP0816	2 2	2 2	1 2	1 2	2 2	1 2	1 2	1 2	2 2	2 2	2 2	
LUP0817	2 2	2 2	2 2	2 2	2 2	2 2	2 2	2 2	2 2	2 2	2 2	
LUP0818	2 2	2 2	1 2	2 2	2 2	1 2	1 2	1 2	2 2	2 2	2 2	
LUP0822	2 2	2 2	1 2	2 2	2 2	1 2	1 2	1 2	2 2	2 2	2 2	
L2	0 0	2 2	1 1	0 0	2 2	2 2	2 2	2 2	0 0	0 0	0 0	**IBH**
43i_2	1 2	2 2	1 1	1 2	2 2	2 2	1 1	2 2	2 2	2 2	2 2	
57i_2	1 2	1 1	2 2	0 0	1 1	1 1	1 1	1 1	1 1	2 2	0 0	
57i2	1 2	1 2	1 2	1 2	2 2	2 2	2 2	2 2	1 2	2 2	2 2	
58i_2	1 2	1 2	1 2	1 1	2 2	1 2	1 2	1 1	2 2	2 2	2 2	
69i_2	1 2	1 2	1 2	1 2	2 2	2 2	1 2	1 2	2 2	2 2	2 2	
L10	1 2	1 2	1 2	1 1	2 2	1 2	1 2	1 1	2 2	2 2	2 2	
L27_3	1 2	1 1	1 2	1 2	2 2	1 2	1 1	1 1	2 2	2 2	2 2	
L28_2	1 2	1 1	1 2	1 2	2 2	1 2	1 1	1 1	2 2	2 2	2 2	
33i_2	2 2	2 2	2 2	2 2	2 2	2 2	2 2	2 2	2 2	2 2	2 2	
34i	2 2	2 2	1 2	1 1	2 2	1 2	1 2	1 2	2 2	2 2	2 2	
35i	2 2	2 2	1 2	1 2	2 2	2 2	1 2	2 2	2 2	2 2	2 2	
36i_2	2 2	2 2	1 2	1 1	2 2	1 2	1 2	1 2	2 2	2 2	2 2	
38i	2 2	2 2	2 2	1 1	2 2	1 2	2 2	1 2	2 2	2 2	2 2	
41i_2	2 2	2 2	2 2	2 2	2 2	2 2	2 2	2 2	2 2	2 2	2 2	
44i_2	2 2	2 2	1 1	1 1	2 2	2 2	1 1	2 2	2 2	2 2	2 2	
56i	2 2	2 2	1 2	1 1	2 2	2 2	1 2	2 2	2 2	2 2	2 2	
59i_2	2 2	2 2	2 2	1 2	2 2	2 2	2 2	2 2	2 2	2 2	2 2	
60i_2	2 2	1 2	2 2	1 2	2 2	1 1	1 2	1 1	2 2	2 2	2 2	
61i_3	2 2	2 2	1 2	1 2	2 2	2 2	1 2	2 2	2 2	2 2	2 2	
65i	2 2	1 2	1 2	1 2	2 2	1 2	1 1	1 2	2 2	2 2	2 2	
67i_2	2 2	1 2	2 2	2 2	2 2	1 2	1 2	1 2	2 2	2 2	2 2	
68i	2 2	2 2	1 2	1 2	2 2	2 2	1 2	2 2	2 2	2 2	2 2	
L1_3	2 2	1 2	2 2	1 2	2 2	1 1	1 2	1 1	2 2	2 2	2 2	
L12_2	2 2	2 2	1 2	1 2	2 2	2 2	1 2	2 2	2 2	2 2	2 2	
L13_3	2 2	2 2	2 2	2 2	2 2	2 2	2 2	2 2	2 2	2 2	2 2	
L14_2	2 2	2 2	1 2	1 1	2 2	2 2	1 2	2 2	2 2	2 2	2 2	
L15_3	2 2	2 2	2 2	1 1	2 2	1 2	2 2	1 2	2 2	2 2	2 2	
L19	2 2	2 2	1 2	1 1	2 2	1 2	1 2	1 2	2 2	2 2	2 2	
L22_2	2 2	2 2	2 2	1 2	2 2	2 2	2 2	2 2	2 2	2 2	2 2	
L26_3	2 2	2 2	2 2	1 2	2 2	2 2	2 2	2 2	2 2	2 2	2 2	
L29_3	2 2	2 2	2 2	1 2	2 2	1 2	2 2	1 2	2 2	2 2	2 2	
L3_2	2 2	2 2	1 2	1 1	2 2	1 2	1 2	1 2	2 2	2 2	2 2	
L5	2 2	2 2	2 2	1 1	2 2	2 2	0 0	2 2	2 2	2 2	2 2	
L6_2	2 2	2 2	2 2	1 2	2 2	1 2	2 2	1 2	2 2	2 2	2 2	
L7_2	2 2	2 2	2 2	2 2	2 2	2 2	2 2	2 2	2 2	2 2	2 2	
L8	2 2	1 2	2 2	2 2	2 2	1 2	1 2	1 2	2 2	2 2	2 2	
L9_2	2 2	1 2	2 2	2 2	2 2	1 2	1 2	1 2	2 2	2 2	2 2	
LUP0172	2 2	2 2	1 2	1 2	2 2	2 2	1 2	2 2	2 2	2 2	2 2	
LUP0173	2 2	2 2	2 2	1 2	2 2	2 2	2 2	2 2	2 2	2 2	2 2	
LUP0185	2 2	2 2	2 2	2 2	2 2	2 2	2 2	2 2	2 2	2 2	2 2	
4959243170_	1 1	1 1	1 1	1 1	2 2	2 2	1 1	2 2	2 2	2 2	2 2	**LR**
4959243170_	1 2	1 2	1 2	1 2	2 2	2 2	1 2	2 2	2 2	2 2	2 2	
4959243170_	1 2	1 2	1 2	1 2	2 2	2 2	1 2	2 2	2 2	2 2	2 2	
4959243170_	1 2	1 1	1 1	1 1	2 2	2 2	1 2	2 2	1 2	2 2	2 2	
4959243170_	1 2	1 2	1 1	1 2	2 2	1 2	1 1	2 2	2 2	2 2	2 2	
4959243170_	2 2	2 2	1 2	1 2	2 2	2 2	2 2	2 2	1 2	2 2	2 2	
4959243170_	2 2	2 2	1 2	2 2	2 2	1 2	1 2	2 2	2 2	2 2	2 2	
4959243170_	2 2	2 2	2 2	1 2	2 2	2 2	2 2	2 2	1 2	2 2	2 2	
4959243170_	2 2	2 2	1 2	1 2	2 2	2 2	1 2	2 2	2 2	2 2	2 2	
4959243170_	2 2	2 2	1 1	1 2	2 2	1 2	1 2	2 2	1 2	2 2	2 2	
4959243170_	2 2	2 2	1 1	1 1	2 2	2 2	2 2	2 2	1 1	2 2	2 2	
4959243170_	2 2	2 2	1 2	2 2	2 2	1 2	1 2	2 2	2 2	2 2	2 2	
LUP0492_2_2	1 1	2 2	1 1	1 2	1 2	2 2	1 2	2 2	2 2	2 2	2 2	**PHA**
LUP0603_2	1 2	2 2	1 2	2 2	2 2	2 2	1 2	2 2	2 2	2 2	2 2	
LUP0604_2_2	1 2	2 2	1 2	1 2	1 2	2 2	2 2	2 2	2 2	2 2	2 2	
LUP0605	1 2	2 2	1 2	2 2	2 2	2 2	1 2	2 2	2 2	2 2	2 2	
LUP0606_2_2	1 2	2 2	1 1	1 1	1 2	2 2	2 2	2 2	1 2	2 2	2 2	
LUP0419	1 1	2 2	1 2	2 2	2 2	2 2	1 1	1 2	2 2	1 2	2 2	**Sharpei**
NELSON_1	1 1	2 2	1 2	1 2	2 2	2 2	1 2	1 2	2 2	1 2	2 2	
SP001	1 1	1 1	1 1	2 2	2 2	2 2	2 2	2 2	1 2	0 0	2 2	
SP003	1 1	1 1	1 1	1 2	2 2	2 2	1 2	1 2	1 2	1 2	2 2	
SP007	1 1	2 2	1 2	1 2	2 2	2 2	1 2	1 2	2 2	1 2	2 2	
SP009	1 1	1 2	1 1	1 1	1 2	2 2	2 2	2 2	1 2	1 2	2 2	
SP101	1 1	2 2	1 2	1 2	2 2	2 2	2 2	2 2	2 2	2 2	2 2	
SP105	1 1	1 1	1 1	1 2	1 2	2 2	2 2	1 2	1 2	1 2	2 2	
SP109	1 1	1 2	1 2	2 2	2 2	2 2	1 2	1 2	1 2	1 2	2 2	
SP113	1 1	1 2	1 1	1 1	2 2	2 2	1 2	1 2	2 2	1 2	2 2	
SP117	1 1	1 1	1 1	2 2	2 2	2 2	2 2	2 2	1 2	0 0	2 2	
SP209_1	1 1	1 1	1 1	1 2	2 2	2 2	1 2	1 2	1 2	1 2	2 2	
SP224_1	1 1	1 2	1 2	1 2	1 2	2 2	1 2	1 1	2 2	1 2	2 2	
EDNA_1_3	1 2	1 2	1 2	1 2	1 2	2 2	2 2	2 2	1 2	1 2	2 2	
SP006	1 2	2 2	2 2	1 2	2 2	2 2	1 1	1 1	2 2	1 2	2 2	
SP008	1 2	2 2	1 2	1 2	2 2	2 2	1 1	1 2	2 2	1 2	2 2	
SP010	1 2	1 2	1 2	1 2	2 2	2 2	1 2	1 2	1 2	1 2	2 2	
SP011	1 2	1 2	1 1	1 2	1 2	2 2	1 2	1 2	2 2	0 0	2 2	
SP102	1 2	2 2	1 2	2 2	1 2	2 2	1 1	1 1	2 2	1 2	2 2	
SP103	1 2	1 2	1 1	2 2	1 2	2 2	1 2	1 2	1 2	1 2	2 2	
SP104	1 2	1 2	1 1	1 2	1 1	2 2	1 2	1 1	2 2	2 2	2 2	
SP108	1 2	1 2	1 2	1 2	1 2	2 2	2 2	2 2	1 2	1 2	2 2	
SP110	1 2	1 2	1 1	1 2	1 2	2 2	2 2	1 1	2 2	2 2	2 2	
SP112	1 2	1 2	1 1	1 2	1 2	2 2	1 2	1 2	2 2	1 2	2 2	
SP115	1 2	1 2	1 2	1 1	1 2	2 2	1 2	1 1	2 2	2 2	2 2	
SP116	1 2	2 2	2 2	2 2	2 2	2 2	1 1	1 1	2 2	1 2	2 2	
SP207_1	1 2	1 2	1 2	1 2	2 2	2 2	1 2	1 2	1 2	1 2	2 2	
SP208_1	1 2	2 2	1 2	1 1	2 2	2 2	1 2	1 2	2 2	2 2	2 2	
SP211_2	1 2	1 2	1 1	1 1	1 2	2 2	1 2	1 1	2 2	1 2	2 2	
SP212_2_2	1 2	1 2	1 1	1 1	1 2	2 2	1 2	1 1	2 2	1 2	2 2	
SP213_2	1 2	1 2	1 1	1 1	2 2	2 2	1 1	1 1	2 2	1 2	2 2	
SP106	2 2	2 2	1 1	1 1	2 2	2 2	1 1	1 2	2 2	0 0	2 2	
SP107	2 2	2 2	1 1	2 2	2 2	2 2	2 2	1 1	2 2	2 2	2 2	
SP114	2 2	2 2	1 2	1 1	2 2	2 2	1 1	1 1	2 2	1 2	2 2	
SP205_1_2	2 2	2 2	1 2	1 1	2 2	2 2	1 1	1 1	2 2	1 2	2 2	
SP206_1_3	2 2	1 2	2 2	1 2	2 2	2 2	1 2	1 2	2 2	2 2	2 2	
SP210_1	2 2	2 2	2 2	1 1	2 2	2 2	1 1	1 1	2 2	2 2	2 2	
CA07	1 1	2 2	2 2	2 2	2 2	2 2	2 2	1 1	2 2	2 2	2 2	**Wolf**
L09CL_23	1 1	2 2	2 2	2 2	2 2	2 2	2 2	1 1	2 2	2 2	2 2	
L09CL_12	1 2	2 2	2 2	2 2	2 2	2 2	2 2	1 2	2 2	2 2	2 2	
L09CL_16	1 2	2 2	2 2	2 2	2 2	2 2	2 2	1 2	2 2	2 2	2 2	
EEP109_d	2 2	2 2	2 2	2 2	2 2	2 2	2 2	2 2	2 2	2 2	2 2	
L09CL_08	2 2	2 2	2 2	2 2	2 2	2 2	2 2	2 2	2 2	2 2	2 2	
L09CL_14	2 2	2 2	2 2	2 2	2 2	2 2	2 2	2 2	0 0	0 0	2 2	
